# The role of noradrenaline in cognition and cognitive disorders

**DOI:** 10.1093/brain/awab111

**Published:** 2021-03-16

**Authors:** Negin Holland, Trevor W Robbins, James B Rowe

**Affiliations:** 1 Department of Clinical Neurosciences, University of Cambridge, Cambridge CB2 0SZ, UK; 2 Department of Psychology, University of Cambridge, Cambridge CB2 3EB, UK; 3 Behavioural and Clinical Neuroscience Institute, University of Cambridge, Cambridge CB2 3EB, UK; 4 MRC Cognition and Brain Sciences Unit, University of Cambridge, Cambridge CB2 7EF, UK

**Keywords:** noradrenaline, locus coeruleus, neurodegeneration, cognition, dementia

## Abstract

Many aspects of cognition and behaviour are regulated by noradrenergic projections to the forebrain originating from the locus coeruleus, acting through alpha and beta adrenoreceptors. Loss of these projections is common in neurodegenerative diseases and contributes to their cognitive and behavioural deficits. We review the evidence for a noradrenergic modulation of cognition in its contribution to Alzheimer’s disease, Parkinson’s disease and other cognitive disorders. We discuss the advances in human imaging and computational methods that quantify the locus coeruleus and its function in humans, and highlight the potential for new noradrenergic treatment strategies.

## Teaser

Holland *et al*. review the role of the noradrenergic system in regulating cognition. They highlight the involvement of this system in many neurodegenerative diseases, including its contribution to clinical symptoms and its potential as a therapeutic target.

## Introduction

Degeneration of the noradrenergic system is a pathological hallmark of many neurodegenerative diseases including Parkinson’s disease, Alzheimer’s disease, and Huntington disease.^1^ Indeed, pathology in the principal source of noradrenaline, the locus coeruleus (LC), can occur before the loss of other neurotransmitter systems commonly associated with such conditions or cerebral atrophy.^2^ Furthermore, the role of noradrenaline in diverse cognitive processes is well established, including vigilance, attention, and learning and memory.^3-7^ Yet, the degree to which noradrenergic systems contribute to the cognitive and behavioural changes resulting from neurological disease is often under-recognized^8^ despite early research reviews^9^ and more recent work to integrate the evidence into a coherent neurocognitive framework.^10^

In this review we bring together the evidence for a central role of noradrenaline in cognition and cognitive dysfunction in neurodegenerative diseases including Parkinson’s disease, Alzheimer’s disease, Huntington disease, frontotemporal lobar degeneration and multiple system atrophy. We draw on data from rodent and non-human primates, while acknowledging important species differences in the neurobiology of the LC. We review the evidence from recent advances in computational models and imaging of the LC in health and disease, and examine the potential for pharmacological and non-pharmacological treatments based on preclinical and clinical studies. Together, these point the way forward to new therapeutic strategies for selective and non-selective noradrenergic treatments.

## The anatomy and pharmacology of noradrenaline and the locus coeruleus

**Figure 1:**
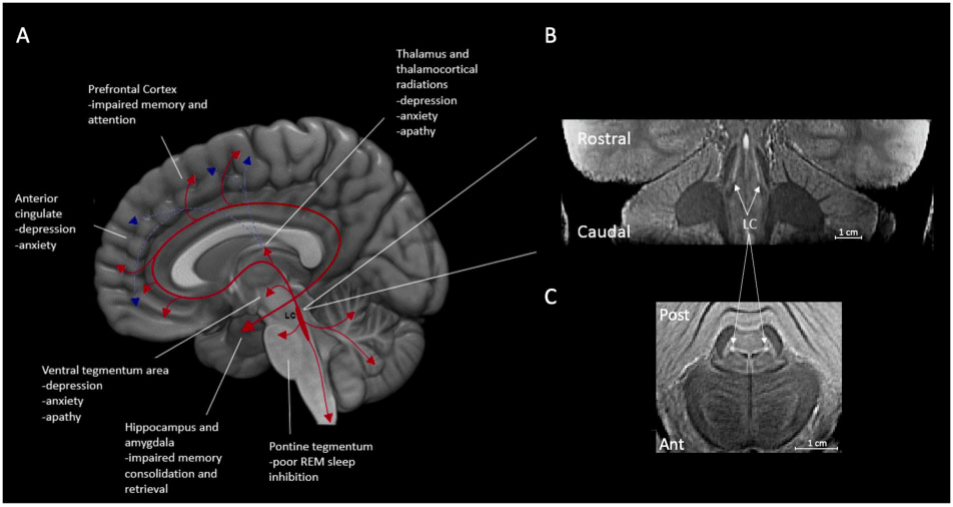
**Neuroanatomical location and projections of the LC**. (**A**) Schematic sagittal view of the brain, illustrating locus coeruleus anatomy, projections, and downstream cognitive dysfunction associated with disturbed LC projections. (**B**) Coronal and (**C**) axial views of the locus coeruleus obtained from magnetization transfer weighted sequences at 7 T MRI. Ant = anterior; Post = posterior. Image courtesy of Dr Rong Ye and Dr Claire O’Callaghan.

The forebrain noradrenergic input is from a small bilateral collection of neurons called the locus coeruleus (LC), where the cells begin rostrally at the level of the inferior colliculus adjacent to the cerebral aqueduct and end caudally near the lateral wall of the fourth ventricle^11^; on axial brain slices, the anatomical landmarks are ∼1 mm under the fourth ventricle, ∼3 mm from the midline, and centred ∼14–21 mm above the ponto-medullary junction^12^ (Fig. 1). The LC is readily identified at post-mortem by its dark colour owing to the high neuromelanin content; the synthesis of the pigment, neuromelanin, is driven by excess levels of catecholamines in the cytosol and is thus crucially linked to the synthesis and metabolism of noradrenaline.^13^^,^^14^ Other conventional ways to identify noradrenaline producing neurons in the LC is by immunohistochemistry directed at tyrosine hydroxylase (TH), the enzyme that converts l-tyrosine to l-DOPA, or against dopamine beta-hydroxylase, which converts dopamine to noradrenaline.^15^ It is widely considered that the two markers are expressed by identical neuronal populations^16^ representing the noradrenergic neuronal population of LC which constitutes more than 95% of neurons in the LC in controls.^17^^,^^18^ However, a small proportion of large TH-positive neurons, which are more numerous in the rostral than caudal pons, lack pigmentation.^15^ Within the LC there is heterogeneity in both the population of the residing medium-sized neurons and neuronal numbers across the rostro-caudal gradient. The majority of medium-sized LC noradrenergic neurons are large multipolar cells (35–45 µm in diameter), with plump cell bodies and short dendrites; in the caudal LC and subcoeruleus, where the density of medium sized neurons is lower, the larger medium sized neurons are interspersed with smaller fusiform noradrenergic neurons (∼15 µm in diameter) with triangular cell bodies and two tufts of long dendrites.^19^ Along the LC, there is a spatially differentiated neuronal organization such that cells giving rise to hippocampal projections are located in more rostral segments while those that innervate the neocortex, cerebellum and spinal cord are located more caudally; subcortical projections of the LC are more scattered, with a proposed spatial bias towards the caudal portion.^14^^,^^19^ The observation of a rostrocaudal gradient has recently been confirmed *in vivo* and is important to consider when assessing for neuronal loss secondary to neurodegeneration where selective loss of rostral or caudal groups may be observed.^20^ We discuss this selective vulnerability later in the review.

The LC is now increasingly studied *in vivo*, through MRI by utilizing the highly paramagnetic neuromelanin content,^21–23^ where the inferior colliculus and the recess of the fourth ventricle are used as key landmarks for its segmentation.^20^ Noradrenaline also arises from the subcoeruleus nucleus extending ventrolaterally from the caudal pole of the LC, innervating the brainstem and hypothalamus for neuroendocrine and autonomic regulation,^11^^,^^24^ but these projections are less relevant for higher cognitive functions.

Given its extensive projections to both cortical and subcortical areas^25^ (Fig.1A), the LC is surprisingly small. With variations in preparation and counting techniques, estimates vary in the range 20 000–98 000 neurons in humans,^26–31^ with the highest estimated neuronal numbers obtained by unbiased stereology.^32^ Within this collection of neurons lie subgroups that preferentially project to the primary motor cortex and the subregions of the prefrontal cortex^33^ leading to a non-uniform release of LC-mediated noradrenaline across the cortical mantle.^34^^,^^35^

**Figure 2:**
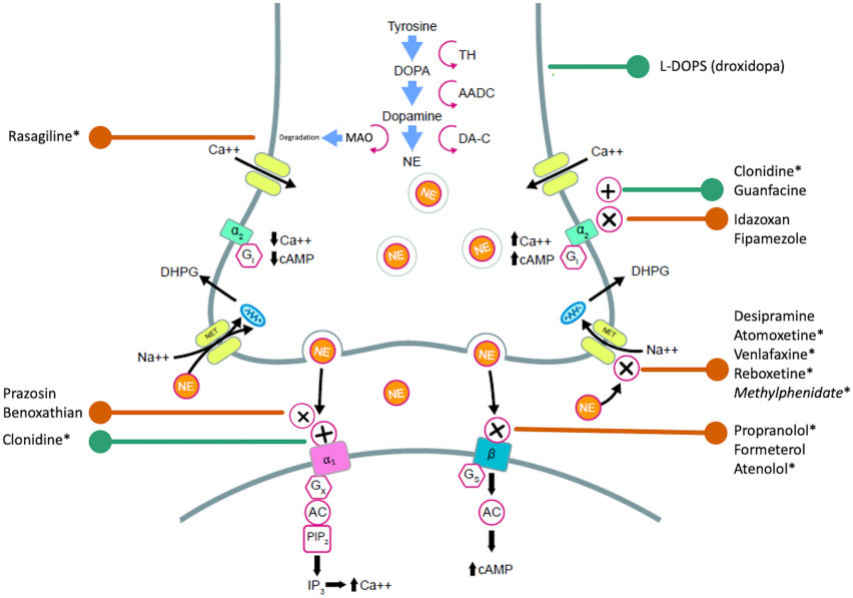
**Noradrenaline synthesis pathway, distribution of pre and postsynaptic adrenoreceptors, and available noradrenergic agonist and antagonists used in animal and human studies.** Agonists are depicted by a plus symbol and dark green arrows, whilst antagonists are depicted by the letter ‘X’ and orange arrows. Drugs used in human studies and clinical trials are marked with an asterisk. Noradrenaline synthesis pathway: noradrenaline is synthesized from tyrosine, which is initially converted to l-DOPA through the action of tyrosine hydroxylase (TH); l-DOPA is further converted to dopamine by aromatic l-amino acid decarboxylase (AADC), before finally being converted to noradrenaline through the action of dopamine β-monooxygenase (DA-C; also known as dopamine β-hydroxylase). Noradrenaline is recycled through the norepinephrine transporter (NET) and degraded by monoamine oxidase (MOA), to the principal end product vanillylmandelic acid or a conjugated form of 3-methoxy-4-hydroxyphenylglycol (MHPG). Methylphenidate = mixed noradrenaline and dopamine reuptake inhibitor.

**Figure 3:**
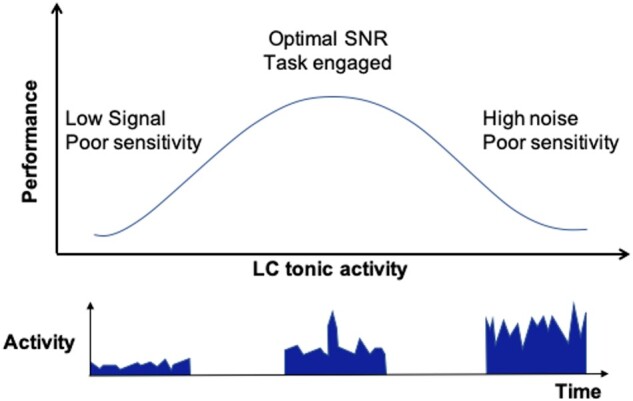
Schematic illustration of the non-linear function of performance versus locus coeruleus activity, analogous to the Yerkes-Dodson model of arousal and comparable to non-linear relationships in dopaminergic and serotonergic systems.

Three factors contribute to the sophistication of noradrenergic transmission. First, the diversity of noradrenergic receptors (Fig. 2). Second, the distinction between tonic and phasic neurotransmission. Third, the non-linear relationship between innervation and performance (Fig. 3).

Noradrenaline exerts an excitatory action through the post-synaptic α1 and β adrenoceptors, and an inhibitory action through mainly presynaptic α2-adrenoreceptors.^36^ The distribution and affinity of adrenoreceptors is highly variable. For example, α2-adrenoreceptor are common in the prefrontal cortical areas, and noradrenaline has the highest affinity for these,^37^ and lower affinity for α1- and β-adrenoreceptors.^38^ As a consequence moderate levels of noradrenaline engage α2 receptors whilst higher levels (released during stress for example) engage the lower-affinity α1 and β receptors.^39^ This creates a non-linear relationship between noradrenergic transmission and performance, indicating that response to an excitatory input may be enhanced or suppressed depending on the receptor in action.^40^

The LC exhibits two broad firing patterns: tonic and phasic (Fig. 3).^41^ These have distinct properties and signal processing characteristics. For example, during direct physiological recordings in monkeys, in a visuo-motor task with reward and punishment, phasic responses followed salient stimuli but not distractors.^41^ Phasic responses were diminished or absent in poor performance trials suggesting a role as an attentional filter that selects for the occurrence of task-relevant stimuli. When not engaged in task performance the LC returns to a tonic firing rate. Within the same visuo-motor task, elevated tonic LC activity reduced the ability to discriminate stimuli from distractors; the monkeys were more distractible and made more errors. These observations are replicated in rats where stimulating LC tonic activity leads to increased decision noise and reduced task participation.^42^ The balance between tonic and phasic activity therefore, enables a gating signal function that regulates task engagement or disengagement according to salience and anticipated rewards or punishments, facilitating an adaptive behaviour. This is supported in human studies, where pupillometry has been used as a surrogate for LC activity^43^^,^^44^ such that a large baseline pupil diameter implies LC phasic activity and a smaller one implies tonic LC activity; for example, in an auditory discrimination task, phasic pupillary dilatation correlated with correct responses, whereas tonic pupillary dilatation correlated with periods of low reward value.^45^ In signal processing terms, dynamic LC activity regulates signal-to-noise ratio both at the level of the LC^46^ and at target neurons.^47^

Histological studies of the LC suggest that neuronal number and volume do not change significantly with age^30^^,^^48^; however, there is an increase in the LC neuromelanin content, which may reflect functional changes that contribute to variability in cognitive performance between healthy young and older adults.^49^^,^^50^ Until recently, studying the LC required invasive methods, limited mainly to preclinical models, or relied on indirect inferences based on the pupillometric response which is correlated with the activity of other neural networks besides the LC. However, advances in neuroimaging, by drawing on the paramagnetic features of the neuromelanin rich LC neurons, have aided the direct *in vivo* study of this structure and its functional connections in humans.

Using MRI enables both *in vivo* human quantification of LC size and neuromelanin content (Fig. 1B and C), and its functional connectivity, with good reliability.^22^^,^^51^ Better resolution and sensitivity of such sequences is being developed alongside post-mortem validation of the histological changes underlying each MRI contrast.^21^ Already, MRI has contributed to understanding the role of the LC in human cognition. For example, in a reversal learning task healthy adults (aged 65–84) were asked to make choices from single or double picture trials with reward and loss as feedback; this was then followed by MRI and a memory test of the pictures seen prior to the scanning session. Those adults with a higher LC signal intensity, performed better at the memory task especially for stimuli associated with negative feedback compared to younger adults (aged 20–31).^49^ A negative correlation has been proposed, between age and the connectivity between LC and ventral tegmental area, and a more complex non-linear relationship between age and the connectivity of LC to frontotemporal cortex.^52^ In the next section, we consider how these properties of the noradrenergic system are affected by neurodegenerative disorders.

## Locus coeruleus neuronal loss in neurodegenerative diseases

LC neuronal loss is common and an early feature of neurodegenerative diseases. Post-mortem studies confirm that LC neuronal numbers are severely reduced in Alzheimer’s disease, Parkinson’s disease, and progressive supranuclear palsy (PSP) to a larger extent compared to neuronal loss in the nuclei commonly associated with these disorders.^53^Table 1 summarizes human studies reporting LC neuronal loss in the neurodegenerative diseases covered below.

**Table 1:** Summary of human studies reporting evidence for LC pathology and noradrenergic deficiency in neurodegenerative diseases

LC pathology/neuronal loss	Study type
**Alzheimer’s disease**	
Neuronal loss in mild cognitive impairment^54^	Post-mortem stereology
Selective loss of middle/rostral LC projections^55^^,^^48^	Post-mortem stereology
Neurofibrillary tangle accumulation within the LC^56^	Post-mortem immunohistochemistry
Reduced noradrenaline transporter PET radioligand uptake within the LC^57^	Post-mortem autoradiography
LC neuronal loss correlates better with illness duration^2^^,^^28^	Post-mortem immunohistochemistry and stereology
Progressive LC neuronal loss with disease progression^48^^,^^58^	Post-mortem stereology
LC signal intensity on MRI correlates with CSF Alzheimer’s disease biomarkers^59^	*In vivo* neuroimaging (MRI) and biochemistry
**Parkinson’s disease**	
Lewy body accumulation within the LC^2^^,^^24^^,^^53^	Post-mortem immunohistochemistry
Lewy body pathology within the LC preceding that within the SN^60^^,^^61^	Post-mortem immunohistochemistry and stereology
Loss of LC neurons more severe at post-mortem than in the SN^62^	Post-mortem immunohistochemistry and stereology
Lower LC signal intensity on MRI in Parkinson’s disease patients with cognitive impairment^63^	*In vivo* neuroimaging (MRI)
Progressive LC signal loss with disease progression^64^	*In vivo* neuroimaging (PET)
**Huntington’s disease**	
LC neuronal loss correlating with disease duration and severity of cognitive impairment^65^	Post-mortem immunohistochemistry and stereology
**FTLD syndromes**	
Progressive supranuclear palsy	
LC neuronal loss and tau accumulation within the LC^27^^,^^66^^,^^67^	Post-mortem immunohistochemistry and stereology
LC neuronal loss negatively correlates with disease severity^27^	Post-mortem immunohistochemistry and stereology
Frontotemporal dementia	
Tau accumulation within the LC^68^	Post-mortem immunohistochemistry
Preserved LC neuronal density^69^	Post-mortem immunohistochemistry
Reduced noradrenaline breakdown products^70^^,^^71^	CSF biochemistry; post-mortem high-performance liquid chromatography
**Multiple system atrophy**	
LC neuronal loss^72^^,^^73^	Post-mortem immunocytochemistry

FTLD = frontotemporal lobal degeneration; SN = substantia nigra.

### Noradrenergic loss in Alzheimer’s disease and related disorders

Alzheimer’s disease is one of the commonest neurodegenerative disorders, characterized pathologically by abnormal tau accumulation in neurofibrillary tangles and the presence of extracellular plaques rich in amyloid-β deposition, and clinically by progressive memory loss and behavioural changes.^74^ Alzheimer’s disease has often been considered as a disorder of cholinergic dysfunction from degeneration of the nucleus basalis of Meynert (nbM)^75^; but there is significant LC neuronal loss which is more severe and better correlated with the duration of illness compared to the nucleus basalis^2^^,^^28^; and there may be selective loss of LC neurons projecting to the hippocampus, from the middle/rostral part of the LC.^48^^,^^55^ Alzheimer’s disease pathology is likely multifactorial and may lie in the interaction between noradrenaline (initiated from the LC) and acetylcholine (initiated from the nbM). This occurs at multiple levels:

(i)LC provided noradrenergic input to the nbM where dopamine beta-hydroxylase immunoreactive terminals make close contact with the choline acetyltransferase (ChAT) immunoreactive neurons.^76^^,^^77^
(ii)High densities of α2-adrenoreceptors are located in the basal forebrain of rat brain^78^ and α2-adrenoreceptor agonists inhibit the release of acetylcholine from the nbM.^79^ For example, infusion of the α2-adrenoreceptor antagonists dexefaroxan or idazoxan increase, whilst agonists at this receptor reduce the release of acetylcholine in the medial prefrontal cortex of conscious rats. The increase in acetylcholine response to dexefaroxan is diminished by noradrenergic depletion in response to DSP4 (a selective LC neurotoxin).^80^ Isolated saporin lesions of the cholinergic system, have been reported to cause little memory impairment despite a 90% loss in the cholinergic neurons in the basal nucleus and 90% of cortical ChAT^81^ while dual lesioning of the noradrenaline and cholinergic systems produce marked cognitive deficits.^82^
(iii)α1- and β-adrenoreceptors mediate excitatory effects of noradrenaline in basal forebrain cholinergic neurons in guinea pigs.^83^

Noradrenaline may also play an important neuro-inflammatory moderator role in the pathogenesis of Alzheimer’s disease via microglial activation. It negatively regulates the transcription of inflammatory genes in astrocytes and microglia, which both express adrenergic receptors.^84^ Lesions of the central noradrenergic systems, including LC pathways, using DSP4 in APP transgenic mice (TgAPP) that are subsequently injected with amyloid-β_1–42_, show an increased number of plaques in response to noradrenergic depletion, together with increased glial activation, reduced mRNA level for the amyloid-β degrading enzyme (metallopeptidase neprylisin, NEP) and reduced plaque degradation.^85^ Indeed, using molecular dynamics simulation, the addition of noradrenaline is shown to inhibit aggregation of amyloid-β_1–42_, and to promote disaggregation of amyloid-β protofibrils.^86^ Furthermore, in a multi-tracer PET imaging study in APP23 transgenic mice treated with DSP4, noradrenaline suppresses microglial transcription of pro-inflammatory genes, and cytokine and chemokine production, as well as regulating phagocytosis and microglia migration, leading to depletion of amyloid-β plaque burden.^87^^,^^88^

Pharmacological manipulation of the noradrenergic system in mouse models of Alzheimer’s disease provides some promise towards therapeutics. For example, treatment of the pro-inflammatory FAD mouse model [with combined mutations in the *App* gene and *Psen1* (presenilin 1) gene] with droxipoda (a precursor for noradrenaline) and atomoxetine (reuptake inhibitor) improves learning in the water maze task. Similarly, treatment of this mouse model with vindeburnol (a derivative of the plant alkaloid vincamine), is associated with reduced inflammation in the LC, and increased noradrenaline levels from an increase in TH activity (the rate limiting step in noradrenaline synthesis); this experiment further shows these mutant mice have reduced anxiety-like behaviour associated with a reduction in amyloid burden in the hippocampus.^89^ However, the response to pharmacological modulation is highly dependent on baseline noradrenergic levels (Fig. 3). For example, stress-induced activation of β-adrenoreceptors in mouse models of Alzheimer’s disease causes further cognitive deficits with greater tau deposition and amyloid accumulation.^90^ The effects of pharmacological enhancement of noradrenaline, however, is yet to be fully evaluated.

These preclinical models are directly relevant to clinical studies. In humans, degeneration of LC neurons occurs in patients with mild cognitive impairment^54^ with accumulation of neurofibrillary tangles,^56^ and a linear progression of LC neuronal loss as the disease progresses into the advanced Braak stages.^28^^,^^48^^,^^58^ Additionally, there is reduced uptake of the noradrenaline transporter radioligand (S, S)-^18^F-FMeNER-D2, in the LC and thalamus of patients with Alzheimer’s disease compared to age-matched controls, which correlates with Alzheimer’s disease severity.^57^ More recently, Betts and colleagues^59^ have illustrated a negative association between the intensity of the LC signal on MRI and levels of amyloid-β in the CSF of patients with Alzheimer’s disease.

Alzheimer’s disease is prevalent in patients with Down syndrome (Trisomy 21), and indeed LC neuronal loss and subsequent noradrenergic deficit is seen in both human and mouse models of this condition.^91^ Patients with Down syndrome and Alzheimer’s disease and those with Down syndrome who subsequently develop Alzheimer’s disease, have lower plasma levels of noradrenaline breakdown products (MHPG; 3-methoxy-4-hydroxyphenylglycol), which significantly correlates with behavioural and psychological signs and symptoms of dementia.^92^^,^^93^ The transgenic mouse model of Down syndrome, Ts65n, exhibits an age-dependent reduction in noradrenaline concentration with poor contextual learning and memory which is partially reversible by droxipoda (noradrenaline prodrug). Treating these mice with formoterol (selective β2-adrenoreceptor agonist), leads to an improvement in contextual learning, reduced hyperactivity, and restored synaptic density.^94^ The use of such drugs in a clinical setting is limited by the abundance of β-adrenoreceptors in cardiac muscles and the associated cardiovascular abnormalities in patients with Down syndrome. More selective, direct manipulation of LC neurons is possible in mouse models using designer receptors exclusively activated by designer drugs (DREADDs). For example, stimulation of designer receptor hM3Dq by clozapine-*N*-oxide reduces hyperactivity and improves novel object recognition, comparable to the effect of droxipoda.^95^ Noradrenaline stimulation may therefore serve as a potential pathway in improving memory loss in selective individuals with Down syndrome and dementia.^96^ Although human literature is limited, in combination with preclinical evidence, it suggests a potential role for adjunctive noradrenergic therapy for selective patients with mild cognitive impairment and Alzheimer’s disease.

### Noradrenergic loss in Parkinson’s disease

Parkinson’s disease is the second most common neurodegenerative disease, characterized pathologically by the presence of intraneuronal α-synuclein containing Lewy bodies^97^; clinically it presents with a characteristic movement disorder typically manifesting as bradykinesia, rigidity, tremor and postural instability^98^ and later cognitive decline.^99^ Lewy body pathology, the hallmark of Parkinson’s disease, occurs prominently in the substantia nigra pars compacta but it is also found in the LC and is associated with cell loss.^2^^,^^24^^,^^53^ Indeed, pathology in the LC is proposed to precede Lewy body formation in the substantia nigra pars compacta,^60^^,^^61^ suggesting that noradrenaline dysfunction may precede dopaminergic deficits in patients with Parkinson’s disease. Translational models of Parkinson’s disease also affect the LC. For example, in the A53T transgenic mouse model, expressing a mutant form of α-synuclein, reduction in noradrenaline but not dopamine is associated with synuclein pathology in the aged mice,^100^ suggesting that α-synuclein pathology is sufficient to cause noradrenergic deficiency. Such animal models also manifest cognitive dysfunction including learning and memory deficits that precede motor deficits,^101^ albeit confounded by anxiety-like behaviour. Pharmacological lesions induced by MPTP (1-methyl-4-phenyl-1,2,3,6-tetrahydropyridine) in non-human primate models, cause both dopamine and noradrenergic deficits,^102^ in association with deficits in attention, attention set-shifting, working memory, cognitive flexibility, and problem solving.^103^

Human studies echo the above findings. The loss of neuronal density within the LC is greater in post-mortem cases of incidental Lewy body pathology compared to the neuronal loss in the substantia nigra,^62^ and dopamine beta-hydroxylase activity and the noradrenergic metabolite dihroxyphenylglycol (DHPG), are both reduced in the CSF.^104^ Similar findings are reported by neuroimaging studies. In patients with Parkinson’s disease with and without mild cognitive impairment, the LC signal on MRI is lower compared to age-matched controls, and is negatively associated with performance on the Trail Making Test B (a test of accuracy and cognitive flexibility).^63^ Using PET imaging in Parkinson’s disease patients exhibiting REM sleep disorder, Sommerauer and colleagues^105^ show a reduced uptake of ^11^C-MeNER (a PET reboxetine analogue with a high specificity for the noradrenaline transporter) in the LC compared with control. Markers of LC functional activity are also reduced in patients with Parkinson’s disease. In a longitudinal study of patients with early Parkinson’s disease, using ^18^F-DOPA PET imaging (a marker of amino acid decarboxylase activity; Fig. 2) Pavese *et al*.^64^ showed significant decline in the region of the LC over 3 years.

Similar to the pathogenesis in Alzheimer’s disease, LC impairment secondary to α-synuclein may also, in part, be immune mediated. T lymphocytes from patients with Parkinson’s disease recognize α-synuclein pathology, and neuromelanin containing organelles within the LC express human leukocyte antigens, leading to antigen presentation of endogenous or exogenous proteins to CD8 positive T lymphocytes; the latter in turn leads to cell death.^106^ The concept of an immune-mediated pathogenesis in Parkinson’s disease has led to the start of clinical trials targeting this pathway.^107^

### Noradrenergic loss in Huntington’s disease

Huntington’s disease is a neurodegenerative disorder caused by unstable expansion of CAG repeats in the huntingtin gene resulting in pathology within both cortical and subcortical areas; clinically it manifests with motor, cognitive and psychiatric symptoms.^108–111^ Dysregulation in the monoamine oxidase A (MAOA) activity, which has a substrate selectivity for noradrenaline and serotonin, contributes to the common symptoms of depression and anxiety in this condition. In studying the YAC128 transgenic mouse model of Huntington’s disease, Garcia-Miralles *et al*.^112^ showed that treatment with clorgyline, restored noradrenaline levels and improved anxiety symptoms and behavioural manifestations of depression; targeting this pathway may therefore be a potential therapeutic option for addressing depression in this disease.

Data from human studies are limited. Post-mortem brains from patients with Huntington’s disease show lower LC neuronal counts associated with features of advanced disease, duration of illness, severity of dementia and impairment in activities of daily living.^65^ However, treatment of 20 patients with mild disease with atomoxetine did not improve performance on a battery of neuropsychological tests.^113^ This clinical study only included patients in the early stages of disease, and data from Parkinson’s disease literature suggests that premature noradrenergic treatment is likely to be ineffective.^114^

### Noradrenergic loss in frontotemporal lobar degeneration syndromes

Several clinical disorders are associated with frontotemporal lobar degeneration, including PSP, corticobasal syndrome, motor neuron disease and frontotemporal dementia. They differ in the degree of evidence for a noradrenergic impairment.

PSP is a neurocognitive disorder, pathologically associated with accumulation of hyperphosphorylated 4-repeat tau, initially within the brainstem and basal ganglia before spreading to the cortex^67^; clinically it typically manifests with a movement disorder including axial rigidity, supranuclear gaze palsy and postural instability, with a dysexecutive, disinhibited and apathetic cognitive profile.^115^ Cognitive deficits in PSP are common and debilitating both for patients and carers. Fifty per cent of patients with PSP present with cognitive symptoms,^116^ and the majority will have a form of dementia during the course of their illness.^117^ The pathophysiology of impairment is multifactorial, but includes impairments of the noradrenergic system: post-mortem studies show PSP-related tau pathology in the LC, and LC neuronal loss,^27^^,^^66^^,^^67^ with α2-adrenoreceptor loss compared to age-matched controls.^58^ Moreover, the loss of pigmented LC neurons are negatively correlated with disease severity as measured by the PSP rating scale.^27^ Pharmacological manipulation in PSP has thus far focused on changing motor symptoms and not so much cognition^118^^,^^119^; however, given the prevalence of cognitive dysfunction, and beneficial effects of noradrenergic modulation in Parkinson’s disease,^120^^,^^121^ further research in this field is required.

Frontotemporal dementia is a clinical syndrome characterized by changes in personality, behaviour and language.^122^^,^^123^ It was formerly known clinically as Pick’s disease, although this term is now generally reserved for the particular pathological entity with 3-repeat tau aggregation (Pick’s disease).^124^ Other causes of frontotemporal dementia include TDP-43 inclusions or fused-in-sarcoma (FUS) pathology starting in but not limited to the frontal and temporal lobes and extending to include the rest of the brain with disease progression.^125^ Frontotemporal dementia lies phenotypically on a spectrum with motor neuron disease.^126^ The degree of change to the noradrenergic system in frontotemporal dementia and motor neuron disease remains unclear.^127^ Characteristic 3-repeat tau accumulation in Pick’s disease and TDP-43 inclusions have been reported in the LC of patients and the mSOD1 transgenic mouse model,^68^ although LC neuronal density may be preserved.^69^ Disruption in the noradrenaline breakdown (MHPG) product have been observed, albeit inconsistently, in the hippocampus, amygdala and frontal cortices of post-mortem of patients with behavioural variant frontotemporal dementia,^71^^,^^128^ and is reported to correlate with emotional lability, dementia severity and agitation.^70^^,^^129^ Pharmacological manipulation of the noradrenergic system may help with the common symptom of impulsivity in this patient group. In a within-subjects, double-blind placebo-controlled study of methylphenidate, a mixed dopamine/noradrenaline reuptake inhibitor, patients with frontotemporal dementia became less risk-taking in the Cambridge Gambling Task, and therefore less impulsive; of course, methylphenidate does not have a pure pharmacological target; however, the study provided some evidence for the role of noradrenergic modulation in this patient cohort.^130^

### Noradrenergic loss in multiple system atrophy

Multiple system atrophy is a progressive neurodegenerative disease associated with oligodendroglial cytoplasmatic inclusions consisting of misfolded α-synuclein affecting the olivopontocerebellar and striatonigral systems.^131^^,^^132^ Clinically it presents with autonomic failure, parkinsonism and/or ataxia,^133^ with cognitive impairment in a minority of patients.^134^^,^^135^ The noradrenergic system in MSA is studied mainly in the context of autonomic dysfunction—a pathological hallmark of this disease.^136^^,^^137^ Loss of noradrenaline in both the LC and the caudal ventro-lateral medulla in patients with MSA has been reported,^72^ and post-mortem studies have shown severe loss of A5 noradrenergic neurons of the pontine tegmentum (projecting onto the medulla and spinal cord), comparable with that seen in the LC in these patients.^73^ Indeed in an open label study of patients with multiple system atrophy and postural hypotension, treatments with droxipoda significantly improved symptoms.^138^ In a recent interventional study in patients with autonomic failure secondary to multiple system atrophy, droxipoda was associated with irritability, confusion and memory impairment that improved on dose reduction or discontinuation; of note, however, none of the participants in this study had any background cognitive impairment on enrolment suggesting a baseline-dependent cognitive modulation in these patients.^139^

## Towards new therapeutic approaches for cognition

Early comparative studies of the role of noradrenaline in cognition drew on two main paradigms: 6-hydroxydopamine (6-OHDA) lesions of the LC or the dorsal noradrenergic ascending bundle to the cerebral cortex and hippocampus; and pharmacological manipulations of adrenoreceptors using agonists (e.g. clonidine) or antagonists of presynaptic inhibitory autoreceptors (e.g. idazoxan). Later, these have been complemented by optogenetics,^140^ and other non-invasive measures. In humans, more recently the principal methods for the study of the noradrenergic system is through non-invasive functional MRI imaging with EEG monitoring,^141^ and 3 T and 7 T MRI imaging of the LC,^21^ or a narrower range of systemic pharmacological manipulations with noradrenergic reuptake inhibitors (e.g. atomoxetine, reboxetine). In this section, we will review the evidence for the role of the noradrenergic system in cognitive domains and review how noradrenergic modulation can benefit or deter performance in health and disease.

We draw the reader's attention to the potential limitations of evidence from non-human studies given the substantial changes the LC system and the target neocortex have been through across phylogeny. Both the LC neuronal number, and the size of one of its most important projection targets, the prefrontal cortex, have undergone considerable evolutionary change. The existence and location of the LC is consistent across non-human primates (chimpanzees, gorillas, gibbons, and macaque monkeys), although the number of TH immunoreactive neurons within the LC are increased in humans, out of proportion to the increase in size of the surrounding anatomy such as the medulla.^142^ Furthermore, the prefrontal expansion in humans is disproportionate to the increase in the size of other neocortical areas.^143^ The inhomogeneity of evolutionary development may underly some of the key behavioural differences between human and non-human primates. In addition, there are species differences in the expression of coexisting neuropeptides within the LC, such as galanin and its receptors, between humans and rodents.^144^ The significance of these neuropeptides for human cognition is not fully resolved. In reviewing the evidence for the role of the LC system in the following cognitive domains, one must remain aware of the caveats related to species differences even where homologies appear to exist.

### Attention

The noradrenergic-LC pathway modulates alertness and attentiveness within a dynamic environment with evidence from cortical depletion of noradrenaline in rats,^5^ and lesions of the α2-adrenoreceptors in monkeys.^41^ Rowe *et al*.^145^ showed that systemic treatment with idazoxan (an α2-adrenoreceptor antagonist) impaired non-reversal task shifting but not task acquisition, indicating narrowed attention in lesioned animals. This result was complemented by a later study of effects of 6-OHDA lesions which selectively impaired extra-dimensional set-shifting.^146^ However, the effect of drugs depends on the noradrenergic state of the animal, which is critical for interpreting treatment potential in patients. For example, in saporin lesioned (noradrenaline depleted) rats, increasing synaptic noradrenaline by administering atomoxetine improved set-shifting but tended to impair this cognitive domain in non-lesioned rats.^147^ A similar baseline-dependent noradrenergic modulation of attention in rats is seen with a novel dopamine and noradrenergic modulator, SK609.^148^

The important balance between tonic and phasic stimulation influences the effect of drug interventions. For example, administering clonidine (an α2-adrenoreceptor agonist) to young healthy adults results in longer reaction times, indicating that inhibition of noradrenergic supply secondary to a global reduction in tonic noradrenergic input, especially in the frontal lobes where these receptors are abundant, may lead to reduced general alertness.^149^ This is echoed in the impairment seen in tasks requiring sustained attention (e.g. rapid visual information processing task) in response to clonidine.^150^ Howerver, this effect is reversed by increasing phasic alertness, and perceptual sensitivity through coupling accessory stimuli with task-related stimuli.^149^ In support of this phenomenon, using pupillometry as a surrogate marker of LC activity, Hoffing and Seitz^151^ show that in a task-irrelevant learning paradigm, healthy volunteers better memorize and recall scenes paired with task-relevant targets than distractors, and perform even better when scenes were paired with novel auditory stimuli. The noradrenaline-LC system is therefore involved in attending, or in computational terms improving the signal-to-noise ratio in the presence of novel or unexpected stimuli and enhances performance accordingly.

In Parkinson’s disease, cognitive dysfunction in the form of dementia and attentional deficit are common.^152^^,^^153^ The Cambridgeshire Parkinson's Incidence from GP to Neurologist (CamPaIGN) study revealed that 46% of patients with Parkinson’s disease developed dementia over 10 years,^153^ and indeed manipulating noradrenaline levels with atomoxetine improves attention in this condition (see Table 3 in Kehagia *et al*.^154^). In Alzheimer’s disease prazosin (a postsynaptic α1-adrenoreceptor antagonist) has been studied in a randomized, double-blind, placebo-controlled study of 22 patients with agitation and aggression; results showed improvements on the Brief Psychiatric Rating Scale, Neuropsychiatric Inventory, and Clinical Global Impression of change. A current clinical trial, looking at the effect of formeterol (long acting β2-adrenoreceptor agonist) on cognition in mild to moderate Alzheimer’s disease is underway.^155^ In Huntington’s disease the evidence is sparse; a randomized controlled cross-over study targeting inattention with atomoxetine in 200 patients with Huntington’s disease, did not show a significant improvement (however, results may be confounded by concomitant use of other psychotropic drugs).^113^

### Working memory

Working memory is the ability to transiently store and manipulate information to guide goal-directed behaviour. It depends on noradrenergic function in humans and animal studies.^156–158^ In rats stimulating postsynaptic α1 receptors (through inducing stress using tasks such as the learned helplessness task for example) impairs working memory—an effect that is seen in the fight/flight response, and is shown to be mediated through the activation of the PKC (phosphatidylinositol-protein kinase C) pathway. This effect is ameliorated through pretreatment with an α1-adrenoreceptor antagonist.^39^ In monkeys systemic and locally administered guanfacine,^159^ and clonidine^160^ improve spatial working memory of aged monkeys; similar results are seen in younger monkeys with experimental lesions of the prefrontal cortex or catecholamine depletion that are subsequently treated with clonidine or guanfacine.^161^^,^^162^ To echo this, Gamo and colleagues^163^ have further shown an improvement in a delayed response task in monkeys treated with the noradrenaline reuptake inhibitor atomoxetine.

Some of the early work linking the noradrenergic system to working memory stems from the disturbance to the noradrenergic pathways in patients with Korsakoff’s syndrome—an amnestic disorder resulting from thiamine deficiency found (mainly) in chronic alcohol use. Levels of the noradrenergic breakdown product MHPG are reduced in the CSF of patients with this condition and this reduction significantly correlates with short-term memory impairment in these individuals. Treatment with the alpha-2 receptor agonist clonidine improves mnemonic and attentional deficit in patients, but has the opposite effect in healthy volunteers—likely due to degeneration of presynaptic terminals and therefore a predominantly postsynaptic action in patients.^164^ The negative effect of noradrenergic modulation in young healthy adults is echoed by Coull and colleagues^165^ who illustrate an impairment in tests of visual working memory in healthy young volunteers in response to clonidine.

Working memory is impaired in both Parkinson’s disease and Alzheimer’s disease (reviewed in Zokaei and Husain^166^). In Parkinson’s disease, nearly 60% of patients have mild cognitive impairment with deficits in working memory at diagnosis, with 42% developing dementia, 6–8 years after diagnosis, even in the absence of Alzheimer’s disease pathology.^167^ Preliminary publications from two ongoing clinical trials, targeting mild cognitive impairment in Parkinson’s disease, have shown inconsistent results with droxipoda (NCT02066571), and atomoxetine (NCT01738191),^168^ although final published results are pending. In Alzheimer’s disease manipulating the noradrenergic system with monoamine oxidase (MAO) inhibitors has shown no overall clinical benefits in a Cochrane review and likewise no clear benefit from atomoxetine in a randomized placebo-controlled trial in Alzheimer’s disease.^169^ Given the complex nature of noradrenergic pathology in Alzheimer’s disease, trials investigating the more selective MAO inhibitor, rasagiline (NCT02359552), and also higher concentrations of atomoxetine in conjunction with other therapies are under way.

### Impulsivity and response inhibition

Impulsivity is a complex construct, which includes risky behaviour in relation to reward, inappropriate and premature responses, and impairment of response inhibition. Noradrenaline modulates several of these components, but there is most evidence for its effect on response inhibition such as the stop-signal task that requires cancelation of an initiated action. Efficiency of response inhibition is often quantified by the stop-signal reaction time. It is dependent on the integrity and function of the inferior frontal gyrus (IFG) in humans and rats,^170^ and modulation by noradrenaline.

The complex pharmacology of the noradrenergic system, and its non-linear relationship to performance, determine whether agents that increase synaptic noradrenaline improve or impair response inhibition. In rats, increasing synaptic noradrenaline with atomoxetine or reboxetine typically improves response inhibition^171^^,^^172^ but this effect is more marked in impulsive animals^173^ in support of baseline-dependent effects.

In healthy adults, atomoxetine can enhance response inhibition using the stop-signal paradigm,^174^ in association with dose-dependent enhancement of activity in the right IFG.^175^ In contrast, an alternative form of response inhibition, using the NoGo paradigms, may be worsened by atomoxetine in health,^176^ noting that this type of inhibition is perhaps more closely associated with serotonergic transmission.^177^

Impulsivity may also be viewed as an urgency to make decisions. In a decision-making task, where healthy adults are asked to sample information before making a decision based on either fixed or decreasing (and negative) rewards, propranolol (a β-adrenoreceptor blocker) reduces information gathering compared to placebo. A Bayesian computation model suggests that the observed effect is likely due to increasing urgency to decide^178^; in other words noradrenergic blockade renders healthy subjects more impulsive.

This evidence is directly related to patients with Parkinson’s disease who are more impulsive than healthy controls even in the absence of severe impulse control disorders.^179^ Patients have longer stop-signal reaction times, less stop-related activation in the right IFG, and weaker functional connectivity between the right IFG and striatum compared with control subjects.^114^^,^^180^^,^^181^ In the Parkinson’s disease group, atomoxetine was found to enhance the stop-related right IFG activation, in proportion to disease severity, as well as restoring interactions between the supplementary motor cortex and the right IFG.^120^^,^^121^ These results are echoed in a double-blind randomized placebo-controlled trial of 25 patients with Parkinson’s disease, where atomoxetine improved stopping accuracy on the stop-signal task and reduced reflection impulsivity and risk taking.^154^

Evidence for noradrenergic modulation of response inhibition also comes from genetic polymorphisms of the noradrenergic transporter (NET), which modulates cortical noradrenaline levels.^182^ In 819 adolescents, this polymorphism influenced activity in the right IFG during response inhibition.^183^ Similarly, in a PET imaging study of 20 healthy individuals, using a NET radioligand, there appeared to be an association between higher scores of impulsivity and lower tracer uptake, albeit not statistically significant.^184^ However, genetic variance in metabolism of noradrenergic drugs like atomoxetine may affect its efficacy^185^ and how we interpret results of noradrenergic drug studies in cognition.

### Cognitive flexibility

By regulating the signal-to-noise ratio during information processing, the noradrenergic system controls the maintenance versus shifting of task sets, which in turn affects cognitive flexibility and behavioural adaptation in a changing environment. During instrumental behaviours, stimulus-response associations may change in different ways: they may reverse within a stimulus set (reversal), or they may shift to new stimulus attributes that are orthogonal to the original associations; this shift may occur within the same feature space (intra-dimensional shift) or to a different stimulus dimension (extra-dimensional shift).^157^ The latter, extra-dimensional shift, is most strongly associated with the noradrenergic system,^186^ although the effect of noradrenergic modulation is once again dependent on the adrenoreceptors in action and their location.

Lesions of the noradrenergic bundle have variable effect on set-shifting. In rodents extra-dimensional set-shifting is selectively impaired through lesions of the dorsal-noradrenergic bundle,^146^ as is the case in rats exposed to the α2-adrenoreceptor antagonist, idazoxan.^145^ Moreover systemic infusion of atipamezole (another α2-adrenoreceptor antagonist) improved attentional set-shifting in another rodent experiment and its beneficial effects were blocked by the infusion of benoxathian, an α1-adrenoreceptor antagonist, into the medial prefrontal cortex; infusion of benoxathian alone did not have any effect.^187^ This is further echoed in an experiment by Snyder *et al*.^188^ who show that infusion of corticotropin-releasing factor into the LC improved extra-dimensional set-shifting whilst at higher doses impaired reversal learning. In support of a Yerkes-Dodson inverted U-shaped behaviour for the action of noradrenaline, atomoxetine has been shown to improve set-shifting in rats with noradrenergic lesions but impairs performance in intact rats.^147^ This result is supported further by Cain *et al*.^189^ who show that adolescent rats respond beneficially to a low dose of atomoxetine in a set-shifting task but not to high doses.

Similar mixed results are evident in human studies. In healthy human volunteers, administration of clonidine (α1/2-adrenoreceptor agonist) dramatically impairs extra-dimensional set-shifting in a task related to the Wisconsin Card Sort Test; an effect that was unexpectedly also seen with the α2-adrenoreceptor antagonist idazoxan, and potentiated with the addition of this to clonidine.^190^ This is in contrast to unequivocal effects on performance on a set-shifting task in young healthy male adults in response to the α2-adrenoreceptor agonist guanfacine.^191^ Data from blockade of the β-adrenoreceptor in improving cognitive flexibility is promising^192^; however, drawing conclusions from these results is difficult with the knowledge that such drugs as propranolol and atenolol reduce stress and anxiety levels and may therefore affect performance accordingly. The application to clinical groups with cognitive inflexibility from Alzheimer’s disease, PSP or Parkinson’s disease is warranted, but data are currently lacking.

## Future directions and conclusions

We have presented evidence for noradrenergic loss in both animal models of neurodegenerative disease as well as in humans and the association with cognitive dysfunction seen in many debilitating neurodegenerative conditions. The noradrenaline reuptake inhibitor atomoxetine has proven well tolerated and promising in targeting impulsivity in patients with Parkinson’s disease; however, treatment studies for cognitive dysfunction in other neurodegenerative conditions are sparse. There are limitations to modulating the noradrenergic system in cognition and these include optimising the balance between too much and too little noradrenaline, the lack of specificity for tonic versus phasic activity, targeting the most symptomatic cognitive domain whilst not jeopardizing others, and personalizing treatment with genetic polymorphisms and baseline characteristics in mind.

We propose that pharmacotherapeutics that normalize noradrenaline levels, neurotransmission and signal-to-noise ratio provide useful strategies for enhancing cognitive function in the neurodegenerative conditions discussed. Noradrenergic effects on cardiovascular and respiratory functioning require careful selection and monitoring in trials, and a progress towards more centrally acting agents. Clinical trials must accommodate the marked individual differences in noradrenergic systems, including polymorphisms that affect noradrenaline synthesis or metabolism,^193^ which may also vary between populations in prevalence.^194^ Non-invasive biomarkers that can signal noradrenergic dysfunction could be used to identify and monitor those at risk. Advances in imaging the human LC, genetics, and relevant outcome measures, are leading towards new individualized noradrenergic treatment strategies for cognitive function in neurodegenerative disorders.

## Funding

The authors are funded by the Wellcome Trust (220258), the National Institute for Health Research Cambridge Biomedical Research Centre (BRC-1215-20014) and the Association of British Neurologists (Patrick Berthoud Charitable Trust, RG99368).

## Competing interests

The authors report no competing interests.

## Abbreviations

IFG: inferior frontal gyrus
LC: locus coeruleus
PSP: progressive supranuclear palsy
